# European active surveillance study of women taking HRT (EURAS-HRT): study protocol [NCT00214903]

**DOI:** 10.1186/1472-6874-6-1

**Published:** 2006-01-17

**Authors:** Juergen C Dinger, Lothar AJ Heinemann

**Affiliations:** 1 Center for Epidemiology & Health Research Berlin, Invalidenstrasse 115, 10115 Berlin, Germany

## Abstract

**Background:**

The post marketing safety surveillance program for a drug containing a new chemical entity should assess both, the safety outcomes that relate specifically to the targeted population, as well as those that could potentially be related to special pharmacological characteristics of the drug. Active safety surveillance using valid epidemiological study designs has been proven to be a pertinent and reliable method to approach this endeavor.

**Methods/design:**

The primary objective of the study is to compare incidence rates of serious adverse events in users of all types of newly prescribed oral HRT products. This active surveillance study will assess pertinent cardiovascular outcomes - in particular venous and arterial thromboembolism - and other serious adverse events (SAEs) in new HRT users over a period of several years. One product under surveillance is Angeliq^®^, which contains the novel progestagen drospirenone (DRSP) combined with estradiol. In addition, all other oral combined HRT products with a novel progestagen or estrogen that will be newly marketed during the study period will be studied. These new HRT products will be compared with established HRT products. The combined cohort will include at least 30,000 women recruited in several European countries. At least 90,000 years of observation are expected from the field work which started in early 2002 and will end around 2008. The participating women will complete a baseline survey using a self-administered questionnaire to describe the baseline risk. After 6 months, 12 months, and then on an annual basis, they will fill out a questionnaire in which they record complaints and events during the use of the prescribed HRTs. All adverse outcomes occurring during the observational period will be evaluated.

**Discussion:**

A complete lifetime medical history, individually validated SAEs over time, and a low loss to follow-up rate are essential for a robust safety assessment. Therefore, the lifetime history of diseases and relevant medications will be documented. Reported SAEs will be validated and analyzed. A four level, multi-faceted follow-up process was established to ensure low loss to follow-up rates (e.g., 3–5% after three years of follow up). Multivariate methods will be used to adjust for confounding.

## Background

The safety of a novel drug product containing a new chemical entity should be assessed in an extensive post marketing safety surveillance program. It is also prudent to assess both, the safety outcomes that relate specifically to the targeted population, as well as those that could potentially be related to the special pharmacological characteristics of the novel drug product. Differentiating between the inherent background population risk and a potential incremental risk due to treatment is often challenging. Active safety surveillance using valid epidemiological study designs has been proven to be a pertinent and reliable method to approach this endeavor.

Long-term active safety surveillance of new HRT products has gained particular importance following the intense scientific debate on the risk/benefit profile of HRT products after the publication of the WHI results [1]. The active safety surveillance study proposed in this protocol, the European Active Surveillance Study of Women taking HRT (EURAS-HRT) study, will assess the risk profile of marketed oral combined HRT products, by comparing HRT products that contain novel progestagens with older HRT products. The primary objective of the study is to compare incidence rates of serious adverse events - in particular cardiovascular outcomes - in new users of all types of oral HRT products. This large, multinational, prospective, controlled, non-interventional, long-term cohort study will follow a series of cohorts for a period of seven years. The study is sponsored by Schering AG with an unconditional grant. Study conduct and data analysis are carried out by an independent investigator under the stewardship of an international advisory committee.

One drug product under surveillance in the EURAS - HRT study is Angeliq^®^, which contains a novel synthetic progestagen drospirenone (DRSP) [2,3] combined with estradiol. In addition, all other oral combined HRT products with a novel progestagen or estrogen that will be newly marketed during the study period will be included in this study. These new HRT products will be compared with established HRT products.

As estrogen/progestagen combinations increase the risk for thrombembolism [4], all new drug products that contain a novel estrogen or progestagen should be investigated for their influence on venous and arterial thromboembolic events rates. A large, prospective, controlled cohort study of OC users (EURAS OC Study), which compared DRSP-containing OCs users with other OCs users, demonstrated that DRSP is not associated with an increased incidence for thromboembolic events in OC users [5]. However, because OC users are two to three decades younger than the typical HRT user the results of the OC study can only partially be extrapolated to older age groups.

Furthermore, given DRSP's antimineralocorticoide pharmacological characteristics, specific outcomes such as the risk of hyperkalemia and dysrhythmia, and subsequent serious cardiac problems will be assessed in this study. As secondary objective all other outcomes of the long-term observation will be evaluated.

A long-term observational cohort study, which compares rates of adverse events among users of Angeliq^® ^(and other new HRT products) with those among users of other marketed oral HRTs, is an appropriate design to assess long-term risks and benefits under real-life-conditions of drug utilization, i.e. taking into account all possible interactions with co-morbidity and co-treatment which are common in higher age groups. Potential differences between first-time users of an HRT product and users who switch from one preparation to another will be investigated. Moreover, the potential of greater risk during the early period of use (e.g. the first year) will be carefully monitored.

The pilot phase of this surveillance study was started in early 2002, using a cohort of women who visited European physicians who prescribe HRT. This revised protocol reflects the experiences gained in the pilot phase of the study. Further background information and details are available at the study's website [6].

## Methods/design

### Objectives

The study will monitor utilization practice with particular attention to long-term effects in user cohorts of: 1) Angeliq (and potentially of other novel oral continuous-combined HRT products); 2) other oral continuous-combined HRTs; and 3) other oral HRTs

under routine conditions, all of these in the medical practice of several European countries.

The study will compare incidence rates of serious or unexpected adverse events among users of the three above-mentioned HRT user cohorts to analyze the following:

- *Events that played a key role in earlier risk considerations*, such as myocardial infarction, stroke, sudden death, and venous thromboembolism;

- *Other medically significant events*.

The study is not designed to investigate long-term outcomes such as breast, colon, or endometrial cancer or osteoporotic fractures, on account of the expected small number of cases and relatively short treatment periods. However, all serious or unexpected outcomes will be fully documented, and evaluated. This means that significant adverse events will be analyzed under "real-life conditions", i.e. without influencing the prescription behavior of the physicians or the desires of the women. It is understood that HRTs will be prescribed according to international guidelines.

### Design

Prospective, controlled cohort study of users of HRT with follow-up: one cohort of women starting with or switching to Angeliq^®^, one comparison cohort of other oral continuous-combined HRT users (starter or switcher), and a third cohort of all other marketed oral HRTs (starter or switcher).

### Study participants

The combined cohorts will include at least 30,000 women aged 40 or more years who started or switched to an oral HRT at the time of inclusion in the study.

Following an indication for hormone replacement therapy, the prescribing physicians will determine whether women are eligible to participate. The primary objective is to accrue women in early or late menopause. For some women, postmenopausal status will be unclear and they will be described as "perimenopausal". Perimenopausal women who are prescribed oral HRTs will be included in this study. There will be stratified analyses for women who were clearly menopausal and women for whom this was not clear for various reasons (e.g., treatment). Women newly taking a herbal menopausal remedy are not eligible for inclusion. Different HRT formulations will be distinguished during the analysis.

Additional study inclusion criteria will not be given. Rather study inclusion will be the decision of the individual prescribing physician after explaining the benefits and risks to the woman. This approach supports the aim of the active surveillance study, which is to reflect drug use under real-life conditions. Evaluation of data for certain subgroups such as "HRT switchers" and "perimenopausal women" can be performed later in the analysis. Subgroup analyses for certain age groups will also be considered.

The women will be asked if they are willing to participate after being fully informed about the study. Women who start with hormone replacement or switch from another HRT can be included. For each woman who receives a prescription for the new HRT at a given center, one woman who receives an HRT prescription from each of the other two groups will be selected.

Women who do not consent to a long-term follow-up study, who have contraindications for HRT, or who have language difficulties, will not be eligible.

### Information gathered

#### Exposure data

Exposure is defined - for this surveillance study - as new use of any oral HRT. This includes women who are first time users (starters) or those who switch to a new brand. The exposure group of particular interest is the group using Angeliq^®^, and two other exposure groups (other oral continuous-combined HRT products and all other oral HRT products) have been defined as comparators (see above). Herbal menopausal drugs and SERMS will not be included in the main analysis.

#### Confounding and effect-modifying factors

Factors considered as confounders, effect modifiers, or adjustment variables will include the following: age, smoking, hypertension, increased blood lipids, treated diabetes mellitus, body mass index, cardiovascular diseases (angina pectoris, myocardial infarction, stroke/TIA, venous thromboembolism), and family history of premature cardiovascular events (MI and stroke). If other potential confounders are identified in the analyses they will be adjusted for as appropriate.

*Outcomes: *The outcomes of particular interest are: cardiovascular mortality, incidence rates of myocardial infarction, stroke, and venous thromboembolism.

The relevant information will be gathered by means of a short self-administered questionnaire to be completed by the participating women (including consultation with their physicians if necessary). The baseline questionnaire will contain questions related to the state of health, risk factors (medical history), and confounders, including drug use. The follow-up assessment of the combined cohort will be done after 6 months, 12 months, and then on an annual basis by means of a questionnaire mailed to the cohort members. This follow-up questionnaire concentrates on experience with the newly prescribed HRT. The study will end when the last woman in the study completes the first year of follow-up and the average follow-up has extended for at least three years.

The questionnaires document the following information:

• Baseline: ID number; birthday; date of last menstrual bleeding; artificial menopause (operation); parity/births (number); OC use, age at start and stop, duration of OC use; any HRT use, how many different brands, duration of HRT, how many switches among brands, date of HRT cessation, name of last brand, name of current prescription; smoking status (current, former, never), number of cigarettes now and before cessation, date of cessation; medical history of relevant diseases such as myocardial infarction, arrhythmias (confirmed by ECG), stroke, pulmonary embolism, deep venous thrombosis, varicose veins, fractures, any cancer; family history of myocardial infarction or stroke, VTE, breast cancer; regular use of medicaments (specify); recent blood pressure reading, height/weight.

• Follow-up: ID number; occurrence of new conditions after last contact such as myocardial infarction, stroke, venous thromboembolism; fractures (specify); any cancer (specify), any other severe health problems (specify); hospitalizations (specify); all brand names of HRTs used since last contact, including dates; regular use of medicaments (specify); changes in smoking status; weight/height; other relevant information; personal changes; name of treating physician/hospital to enable contact (if an AE/SAE has occurred).

If the women report serious adverse events (SAEs), the investigators will discuss these events with the women and the treating physician(s). Serious adverse events will be reported to the respective manufacturer regardless of whether the attending physician already reported the event through official channels in the respective country. Necessary steps will be taken to facilitate rapid reporting in the event of unexpected SAEs.

### Data handling

The questionnaire will first be checked manually for completeness and consistency at the coordinating center. In case of missing data, contradictions, questions, or obvious errors, the participants will be contacted for clarification. After completion of manual checks, the forms will be entered into an electronic database designed to meet the specific needs of a long-term cohort study.

Non-respondents will be sent a second, and if necessary, a third mailing. The names and addresses of the remaining non-respondents will then be checked in compliance with national laws to obtain information about possible changes. For any remaining non-respondents, attempts at tracing will be made using the contact persons named at the time of last contact.

To further maximize follow-up after the baseline survey, the women will be asked at every contact to announce any personal changes, e.g. telephone numbers. If a woman reports any of a specified list of medical outcomes, a follow-up form will be sent to request consent to inspect the medical records. Information on the name of the relevant physician, hospital, etc. will be recorded in order to gain access to the records.

### Study size estimation

The follow-up of at least 30,000 women will generate 90,000 or more women-years within the 5–6 year study period.

The sample size calculation is based on estimates for the incidence of important outcomes in this age group and a market share of 10% for the new HRT product(s) of interest: cardiovascular mortality (0.5%), non-fatal myocardial infarction (0.2%), stroke (0.2%), and VTE (0.3%).

To detect an increased risk of certain outcomes associated with new HRT products with 95% probability and a power of 80%, the roughly required number of women-years of the combined cohort should have the following magnitude for a detectable rate ratio of 3.0: cardiovascular mortality about 44,000 women-years, myocardial infarction 30,000, and venous thromboembolism 20,000. Therefore the expected 90,000 women-years should be sufficient to investigate the adverse events of interest.

### Evaluation

Based on *a priori *considerations of the clinical pharmacology of the new continuous-combined HRT Angeliq^® ^and also other HRTs, there is a substantial potential for confounding in this study. It will not be possible to confine the study to non-predisposed women, since an important objective in this study is to determine risk according to the baseline risk status. It will be important to control confounding and to evaluate possible effect modification from various sources. Reported serious adverse events will be validated with medical records. Ascertainment of deaths - in particular sudden death - will be accomplished by cross-reference to relevant death registries (in countries were access to these registries are permitted by national law).

### Interim evaluations

Descriptive statistics of the interim results will be presented twice a year to the Advisory Council, including data on the number of women enrolled in the three user cohorts, observed duration and type of HRT use, number and nature of serious AEs for all three HRT cohorts, and as soon as sufficient numbers are available also stratified by starters and switchers, or - if relevant - by certain formulations or specific events (e.g. sudden death, cardiovascular events, or other major events), provided that sufficient numbers have been obtained or the nature of the events is serious, unexpected, or relevant for the study objectives.

Any adverse events mentioned in the follow-up questionnaires or received as separate reports will be compiled by local investigators and forwarded to the international coordinating center in Berlin. Additional information will be sought as appropriate: Firstly, the reporting patient will be contacted again to confirm the absence of misunderstanding and to acquire more information about symptoms and other circumstances. These include confirmation of name/address of the treating physician. Depending on the results, contact will be made with relevant health professionals to gain access to detailed medical information to confirm the (new) event/diagnosis. The clinical seriousness, causality, and possible association of each reported adverse event with HRT use will be assessed.

For the overall safety assessment, the relevant data as well as conclusions will be submitted to the Advisory Council as part of the next scheduled progress report, or immediately in case of urgency. There may be a need for interim safety reports that can be published after assessment by the AC.

### Final analysis

The primary aim of the analysis is to assess cardiovascular effects; the secondary objective is to evaluate all other outcomes of the long-term observation. A detailed analysis plan was prepared and reviewed/approved by the Advisory Committee in September 2004 and March 2005.

Several issues have to be considered in the analysis and interpretation. For example, it cannot be excluded that women who were prescribed the most recently launched HRT (here Angeliq^®^) will be found systematically to be at higher risk than women who were prescribed older HRTs. In addition, it cannot be excluded that starters (first ever users) of the *new *continuous-combined HRT (Angeliq^®^) have an even higher baseline risk than starters of other recent HRTs due to the physicians' prescription behavior, which is influenced by the expectation of a safer "*new HRT*". Furthermore, women who have previously used older HRTs may be at lower risk than new users, because, on average, these will have used the older HRTs for a longer period of time whereas those who experienced cardiovascular events will no longer be using them. Comparisons between first-time users of HRTs should therefore be easier to interpret.

To adjust for confounding and effect modification, multivariate methods will be used. It is anticipated that there may be complex sources of bias and confounding, e.g. many risk factors could contribute to the relevant outcomes. In interpreting the findings, therefore, the greatest emphasis will be on relative risk estimates of high magnitude. Low relative risk estimates (under 2.0 - 3.0) will be considered inconclusive, because it is methodologically impossible to distinguish between causation, bias, and confounding. Any apparently increased relative risk, if present, will be evaluated based on the magnitude of the observed hazard ratio. In interpreting the impact of results on public health (safety), the main emphasis will be on absolute risk estimates.

The focus will be on prudent analysis with appropriate statistical adjustment procedures across centers and countries. Comparisons among countries are not the aim of the study but could be made on request of the Advisory Council.

The final analysis will be based on life-table methods comparing the three main cohorts of users. All analyses will make allowance for confounding, using methods that will include multivariate techniques such as Cox regression, stratification by the Mantel-Haenszel procedure, and exclusion of women with specific confounders at baseline. Some confounders that can be precisely quantified (e.g. smoking status, obesity) will not be excluded but rather controlled for in the analysis.

### Ethics and privacy

The planning and performance of the study are subject to national laws. The study was only started after meeting all requirements of the appropriate regulatory authorities. It will be conducted in accordance with the ethical principles of the Declaration of Helsinki. The study was approved by the ethical committee in Duivendrecht, The Netherlands (Medisch Ethische Toetsingscommissie of the Stichting Therapeutische Evaluatie Geneesmiddelen) in May 2004. The study is registered in the public clinical trials registry of the US National Library of Medicine under the registration number NCT00214903.

The women participate on a voluntary basis. The women will be asked if they are willing to participate, i.e. to report complaints/symptoms/events for years under the assumption that their data will be kept anonymous and will be evaluated/reported as statistics only. The nature of the study will be explained to each woman prior to her entry into the study, including its purpose and associated procedures as well as the expected duration of the study, i.e. including potential benefits and side effects of HRT. After consenting, the women will receive signed and dated duplicates of the volunteer information and informed consent forms. Each woman will have ample opportunity to ask questions and will be informed about the right to withdraw from the study at any time without any disadvantage and without having to provide reasons for this decision.

Women and treating physicians will be kept anonymous in the scientific database. Names and addresses will be stored under lock and separately from the scientific (questionnaire) data. Protocol and study material were submitted to the relevant Data Privacy Institution. The specifications of the respective data protection laws will be followed.

### Advisory council

An independent Advisory Council (AC) consisting of epidemiologists, gynecologists, cardiologists, and other experts of international reputation is responsible for overseeing the study. The AC met for the first time on 18 February 2004. Names and terms of reference are given in the annex. The AC will continue to meet several times during the study period. Non-voting representatives of the sponsor and the investigator will attend the meetings. At the end of each meeting the AC evaluates the findings in executive sessions without the investigator and the sponsor.

### Study management

The Centre for Epidemiology & Health Research Berlin, an independent scientific institution experienced in international, multicentric observational studies, is responsible for planning and conducting the study in Europe. It will coordinate the fieldwork in different countries and subcontract with local investigators to handle language barriers.

## Discussion

Estrogen/Progestagen combinations increase the risk of venous thromboembolism, as a class. As illustrated by the WHI study on HRT these products increase venous thromboembolism maximally during the first year of use, after which the risk declines (starter effect) [1]. Women who are susceptible to VTE probably experience a thrombosis soon after they start HRT intake and then stop treatment. Therefore, a population of long-term users who do not switch between different preparations includes only a few high-risk patients and therefore has a low VTE rate. The starter effect repeats itself at a lower level after a period of discontinuation (re-starter effect) [7,8]. Furthermore, women at high risk tend selectively to switch to the most recently marketed product (switcher effect). For the sake of the validity of the study results and the comparability of the cohorts it is essential to record precise information on duration of use, discontinuation periods and risk factors. Long-term users of a HRT preparation should not be included in the study.

The diagnosis of venous and arterial thromboembolism is not always confirmed by methods with a high sensitivity and specificity (e.g., phlebography). Therefore, the classification of reported thromboembolic events has to be done by a predefined algorithm. Definite and probable events will be considered as confirmed. All other cases will be classified as "not confirmed".

One of the most crucial factors to ensure the validity of the study is a low "lost to follow-up" rate. In order to minimize loss to follow-up a multi-faceted, four level follow-up process was established. The level1 activities include the mailing of the follow-up questionnaire and - in case a woman does not respond - two reminder letters. If level1 activities do not lead to a response multiple attempts are made to contact her, her husband, friends, relatives and the gynecologist/primary care physician per phone. In parallel to these level2 activities searches in national and international telephone and address directories are started (level3 activities). If this is not successful, an official address search via the respective governmental administration is conducted (in some countries centralized, in others decentralized at community level). This level4 activity can provide information on a new address (or information that the respondent has moved abroad or has died). If necessary, a search in the national death registers could be started at the end of the study to clarify the vital status of patients who are lost to follow-up after level4 activities. Overall, the investigators estimate that the loss to follow-up of the combined cohort could be kept at less than 5% of the recruited population.

## Competing interests

The study is funded by Schering AG, Berlin, Germany, as part of a post-approval commitment to regulatory authorities. The study is scientifically independent and governed by an independent Advisory Council. The members of the AC are remunerated for expenses and receive an honorarium to compensate for potential loss of earnings during their work for the advisory council. The members will not be involved in or paid for the operational conduct of the study.

The Advisory Council is/was responsible for the approval of the protocol, analysis plan, final report, publications as well as the safety monitoring of the (interim) results. The publication manuscript was approved by the Advisory Council on September 26, 2005.

## Authors' contributions

JCD and LAJH contributed equally to the design of the study as well as to the writing and the reviewing of the manuscript.

## Pre-publication history

The pre-publication history for this paper can be accessed here:


